# Interocular Retinal Nerve Fiber Layer Thickness Difference in Normal Adults

**DOI:** 10.1371/journal.pone.0116313

**Published:** 2015-02-13

**Authors:** Seung Woo Hong, Seung Bum Lee, Dong-hyun Jee, Myung Douk Ahn

**Affiliations:** 1 Department of Ophthalmology and Visual Science, College of Medicine, The Catholic University of Korea, Seoul, Korea; 2 Department of Ophthalmology, Armed Forces Capital Hospital of Korea, Seongnam city, Gyeonggi province, Korea; Massachusetts Eye & Ear Infirmary, Harvard Medical School, UNITED STATES

## Abstract

**Purpose:**

To determine the interocular retinal nerve fiber layer (RNFL) thickness difference of normal subjects.

**Methods:**

Both eyes of 230 normal adults received peripapillary RNFL thickness measurements using OCT. The effect of ocular cyclotorsion on the RNFL thickness profile was mathematically corrected. The fractional and absolute interocular RNFL thickness differences at 256 points of peripapillary area were calculated. We divided the subjects into 3 groups according to the locations of superior and inferior peak thickness, respectively, and compared the interocular RNFL thickness differences between the subgroups.

**Results:**

The fractional interocular RNFL thickness difference exhibited smaller regional variations than the absolute interocular difference. The means of fractional interocular differences were 0.100 ± 0.077 in the temporal half area and 0.146 ± 0.105 in the nasal half area, and the tolerance limits for the 95th and 99th distributions were about 0.246 and 0.344 in the temporal half area and 0.293 and 0.408 in the nasal half area, respectively. The fractional interocular differences of subgroups classified by the locations of superior and inferior peak RNFL thickness showed difference at smaller areas than the absolute interocular differences (19 and 8 points versus 49 and 23 points, respectively).

**Conclusion:**

Glaucoma can be strongly suspected, if interocular fractional RNFL thickness difference is over 25% at 5 consecutive points or over 35% at 3 consecutive points in the temporal half area. The fractional interocular comparison is a better diagnostic approach because the fractional interocular RNFL thickness difference is less influenced by the locations of peak RNFL thickness.

## Introduction

Diagnosing glaucoma using OCT usually consists of comparing retinal nerve fiber layer (RNFL) thickness of a patient to the built-in normative RNFL thickness, and the normative RNFL thickness usually has its peak RNFL thickness at the 11 and 7 o’clock position (on the right eye orientation). The comparison to normative RNFL thickness has shown good sensitivity and specificity for diagnosing glaucoma [1–5]. However, in some individuals, the locations of the peak RNFL thickness are so different from that of the normative RNFL thickness that comparing their RNFL thickness profiles to the normative RNFL thickness provides false information. According to previous studies that investigated the relationships between the locations of the peak RNFL thickness and refractive status, myopic eyes with a long axial length are likely to have more temporally located superior and inferior peak RNFL thicknesses in the thickness profile [6–9].

Considering that myopia has an association with glaucoma [10,11], diagnosing glaucoma in patients who have deviated RNFL thickness profiles is very important, even though these patients are a minority. In these cases, interocular comparison of the RNFL thickness can be an alternative diagnostic approach because the RNFL thickness profiles of healthy right and left eyes are generally mirror images of each other. However, organ pairs are not always perfectly symmetric, and little is known about the interocular symmetry of the RNFL. The purpose of the present study was to determine the interocular RNFL thickness difference in normal healthy eyes. We also investigated the difference between the interocular RNFL thickness difference of normal eyes with deviated RNFL thickness profiles and normal eyes with non-deviated RNFL thickness profiles.

## Methods

We recruited 260 individuals who met our eligibility criteria as normal subjects. These individuals were recruited through an advertisement at the Armed Forces Capital Hospital, and the subjects voluntarily contacted the research staff for enrollment. This study was approved by the Institutional Review Board of Armed Forces Capital Hospital and adhered to the tenets of the Declaration of Helsinki. All subjects provided written informed consent.

The exclusion criteria were as follows: best-corrected visual acuity worse than 20/20; anisometropia > 1.5 diopter or interocular axial length difference > 0.3mm; history of ocular trauma including intraocular or refractive surgery; history of any ocular, systemic, or neurologic disease that could affect the RNFL; any presenting ocular pathology capable of causing visual disturbance; closed or occludable angle on gonioscopic examination; intraocular pressure >21 mmHg; evidence of a reproducible visual field (VF) defect in either eye; unreliable VFs (false-positive or false-negative rate >15% or fixation losses >20%);any optic nerve head abnormality including acquired pit (APON) or notching or disc hemorrhage; and any suspicious RNFL defect on RNFL photographs.

Each normal subject underwent a comprehensive ophthalmologic evaluation. Manifest refractions were performed with an autorefractometer (Canon R-F10; Canon Inc., Japan), and VF examinations were performed using the SITA FAST protocol of the Humphrey VF analyzer (HFA II 750–4.1 2005; Carl Zeiss Meditec Inc., Dublin, CA, USA). The axial length was measured using a biometer (IOL Master, Carl Zeiss Meditec Inc.), and the optic disc, fundus, and RNFL were photographed with a digital fundus camera (Canon CF-60UFi; Canon Inc.).

The eyes of the normal subjects who met the eligibility criteria were scanned using the Cirrus HD OCT system (version 4.0.0.64) after pharmacologic pupil dilation. Only scans that did not have movement or blink artifacts within the scan circle and had a signal strength ≥7 were accepted. Unacceptable scans were discarded and new scans were obtained. Among the 3 scans obtained for each eye, the scan with the highest signal strength and least eye movement was selected. The RNFL thickness value at each of the 256 measurement points (0–255) was recorded. The locations of the superior and inferior peaks of RNFL thickness were recorded in point unit.

**Fig 1:**
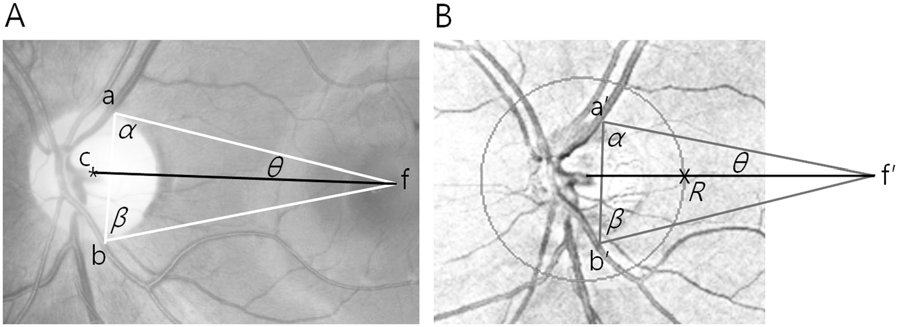
Fundus photograph and retinal nerve fiber layer (RNFL) thickness deviation map showing the new reference lines. The new reference line (black line) on the fundus photograph (A) connects the geographic center of the optic nerve head (point c) and the fovea (point f). The white triangle has corners at the fovea (point f) and the points where the temporal borders of the superior temporal retinal vein (point a) and the inferior temporal retinal vein (point b) cross the disc margin. Side a-f and the line c-f make the angle θ between them. On the RNFL thickness deviation map (B), a similar triangle (gray triangle) was constructed with 2 corners at the points where the temporal borders of the superior temporal retinal vein (point a’) and the inferior temporal retinal vein (point b’) cross the disc margin and side a*-b*. The other corner of the triangle was point f*. Angle θ was measured and a line was drawn to the optic disc (black line). Finally, point *R* was marked where the scan circle and the line met.

The RNFL thickness values were corrected for ocular cyclotorsion because a previous study revealed that correction for ocular cyclotorsion improved the interocular symmetry of RNFL thickness [12] and because regional interocular RNFL thickness difference may vary according to head positioning [13]. Currently, Spectralis OCT (Heidelberg engineering, German) provides “FoDi (Fovea-to-Disc) Alignment Technology” for ocular cyclotorsion correction. However, we performed this correction mathematically, because the Cirrus OCT did not provide the technology. To this end, we drew a line between the geographic centers of the optic nerve head and the fovea, and set the point at which the line crossed the scan circle of the OCT as a new reference point (point 0). Then, we rearranged the RNFL thickness result to the new reference point. Because the RNFL thickness deviation map of Cirrus OCT does not contain the fovea, we drew the reference line on the fundus photograph and moved it to the RNFL thickness deviation map. The procedures were as follows (Fig. 1): we drew a triangle with corners at the fovea and the points where the temporal border of the superior temporal retinal vein and the temporal border of the inferior temporal retinal vein crossed the disc margin on the fundus photograph, and we subsequently measured the angle *θ* between a side of the triangle connecting the fovea and one of the other corners and the reference line. On the RNFL thickness deviation map, a similar triangle was constructed with 2 corners at the points where the temporal border of the superior temporal retinal vein and the temporal border of the inferior temporal retinal vein crossed the disc margin and a side connecting these 2 points. At the other corner of the triangle (corresponding to the fovea), the angle *θ* was measured and a line was drawn in the direction of the disc. Finally, the point was marked where the scan circle and the line met, which was used as the new reference point (point 0). This procedure is explained in detail in our previous study [12].

At each of the 256 points, we calculated the interocular RNFL thickness difference and the fractional interocular difference in RNFL thickness. We defined the fractional interocular RNFL thickness difference as the absolute interocular RNFL thickness difference divided by the larger RNFL thickness value among the 2 eyes {i.e., (the larger RNFL thickness value—the smaller RNFL thickness value)/the larger RNFL thickness value}.

The subjects were classified into 3 subgroups based on the locations of the superior and inferior peaks of RNFL thickness:(1) subjects in whom the peak location point was equal to or lower than the mean-1 standard deviation (SD) in one or both eyes, (2) subjects in whom the peak location points were between the mean-1SD and the mean +1SD in both eyes, and (3) subjects in whom the peak location point was equal to or higher than the mean +1SD in one or both eyes. The interocular RNFL thickness differences and fractional interocular RNFL thickness differences in each group were compared.

Image-editing software (Photoshop CS5, ver. 12.0.1; Adobe Inc.) was used to make drawings and measurements on images. Statistical software (version 13.0, SPSS Corp., IL, Chicago) was used for statistical analyses and plotting the graphs. For all tests, the statistical significance was set at 5% and determined by a *p* value < 0.05. The absolute and fractional interocular differences in RNFL thickness were compared using non-parametric tests, because these differences did not show a normal distribution (P<0.001, Kolmogorov-Smirnov test).

## Results

**Fig 2:**
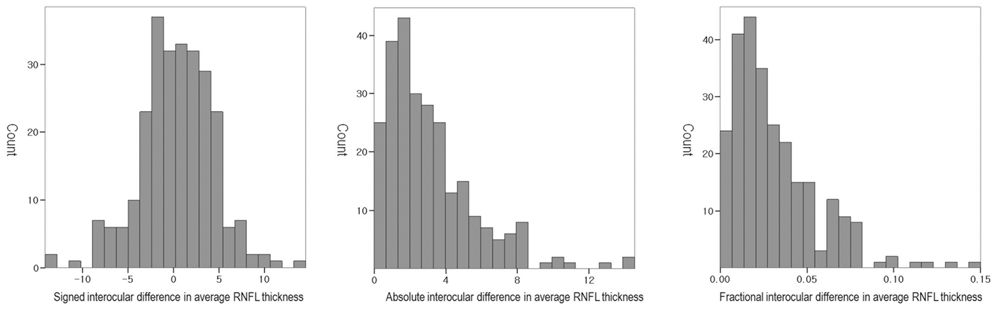
Histograms showing the distributions of the signed, absolute and fractional interocular differences in average RNFL thickness of subjects.

Of the 260 healthy normal subjects in this study, 219 were men (84.23%) and 41 were women (15.77%). All subjects were ethnically Korean and the mean age was 22.38 ± 4.100 years (range, 19–39 years). The new reference line met the OCT scan circle at point 250.39 ± 2.38 in the right eye and at point 251.53 ± 3.21 in the left eye. The ocular information for the study population is summarized in Table 1. The original and corrected locations of the superior peak RNFL thickness were more temporally located in the right eye than in the left (P<0.001, paired t test). The OCT scan signal strength was higher in the right eye than in the left (P = 0.013). No statistically significant interocular differences were observed for the other variables (P>0.05). The distributions of the signed, absolute and fractional interocular difference in average RNFL thickness is shown in Fig. 2.

**Table 1:** Summary of ocular information from normal subjects (n = 260).

Ocular variable	Right eye	Left eye	Signed difference	P value*
Spherical equivalent of refractive error (Diopter)	-2.363 ± 2.221	-2.336 ± 2.206	-0.026 ± 0.506	0.401
Axial length (mm)	24.824 ± 1.180	24.821 ± 1.173	0.003 ± 0.217	0.839
Original location of superior peak RNFL thickness (point)	50.38 ± 6.82	52.90 ± 7.23	-2.523 ± 5.823	<0.001
Corrected location of superior peak RNFL thickness (point)	54.90 ± 6.93	56.74 ± 7.04	-1.838 ± 4.617	<0.001
Original location of inferior peak RNFL thickness (point)	205.49 ± 8.10	206.06 ±7.92	-1.400± 6.600	0.163
Corrected location of inferior peak RNFL thickness (point)	210.01 ± 7.85	209.90 ± 7.75	0.112 ± 5.502	0.744
Average retinal nerve fiber layer thickness (μm)	98.93 ± 8.74	98.53 ± 8.91	0.405 ± 4.007	0.104
Signal strength of scan	8.62 ± 0.97	8.48± 1.07	0.138± 0.890	0.013

Signed difference (right eye minus left eye)

* Paired *t*-test

**Fig 3:**
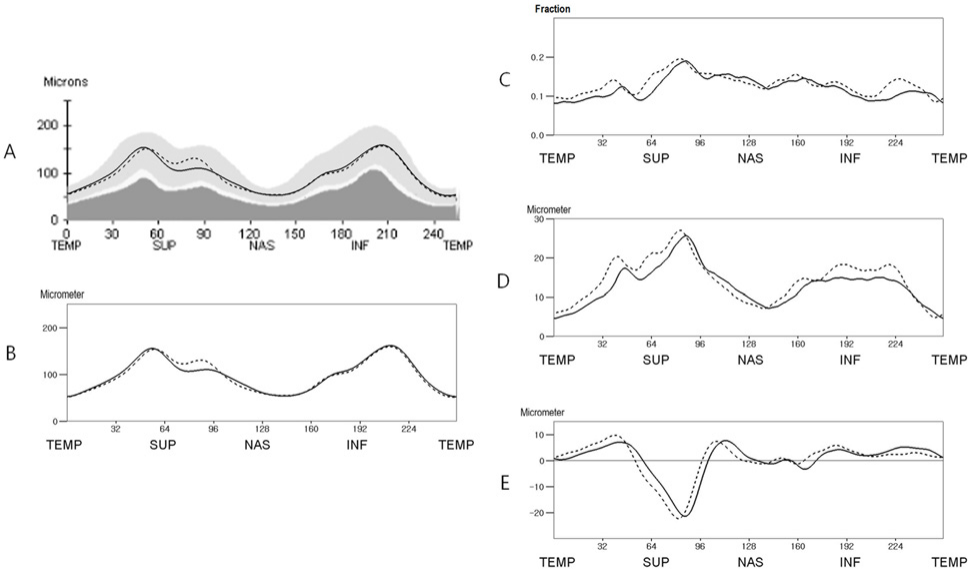
Mean retinal nerve fiber layer (RNFL) thickness and interocular RNFL thickness difference of the study population (n = 260) before and after correction for ocular cyclotorsion. A. The mean RNFL thickness before correction for ocular cyclotorsion. B. The mean RNFL thickness after correction for ocular cyclotorsion. C. The mean fractional interocular RNFL thickness difference. D. The mean absolute interocular RNFL thickness difference. E. The mean signed interocular RNFL thickness difference. (In A and B: straight line, right eye; dotted line, left eye. In C, D, and E: straight line, ocular cyclotorsion corrected value; dotted line, original value)(TEMP = Temporal, SUP = Superior, NAS = Nasal, INF = Inferior).

**Fig 4:**
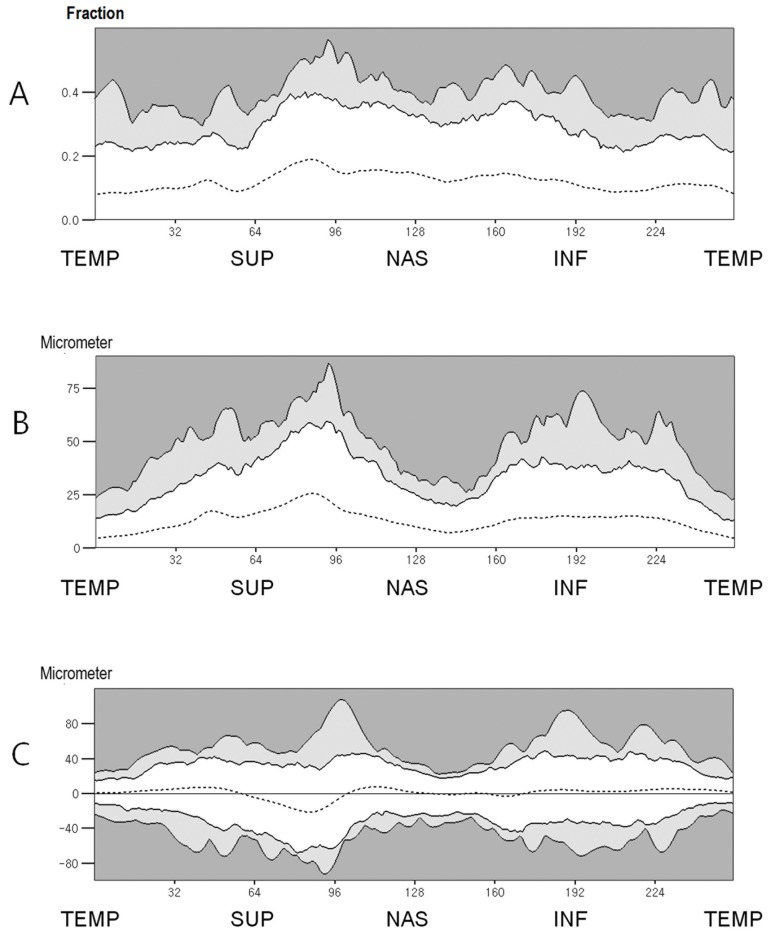
Percentile distributions of interocular RNFL thickness difference. A. The percentile distributions of the fractional interocular RNFL thickness difference. B. The percentile distributions of the absolute interocular RNFL thickness difference. C. The percentile distributions of the signed interocular RNFL thickness difference. (White zone, within the 95th percentile distribution; light gray zone, between the 95th and 99th percentile distribution; dark gray zone, outside the 99th percentile distribution)(TEMP = Temporal, SUP = Superior, NAS = Nasal, INF = Inferior).

The mean RNFL thickness at 256 points are shown in Fig. 3. The absolute and fractional interocular difference decreased by 10.11% and 7.89%, respectively, after correction for ocular cyclotorsion (Fig. 3C and D). Around the 1 to 2 and 5 o’clock positions (right eye orientation; at points 61–98 and 159–168), the left eyes had a thicker RNFL than the right eyes (P<0.05, paired t test: Fig. 3E). Around the 8 to 11, 3, and 6 o’clock positions (at points 0, 10–54, 103–126, 178–198, and 213–255), the right eyes had a thicker RNFL than the left eyes (P<0.05; Fig. 1E). The percentile distributions of the interocular differences are shown in Fig. 4.

**Fig 5:**
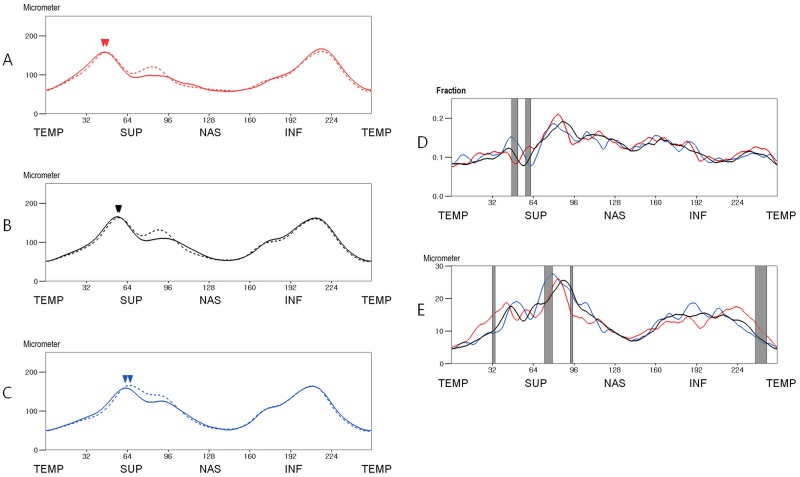
Mean RNFL thickness of subgroups classified by the location of the superior peak thickness after correction for ocular cyclotorsion (A, B and C) and comparison of interocular RNFL thickness differences between the subgroups (D and E). A. Mean RNFL thickness of group 1 (subjects in whom the corrected location point of the superior peak was ≤ 48.78 in 1 or both eyes, n = 44). B. Mean RNFL thickness of group 2 (subjects in whom the corrected location point of the superior peak was between 48.78 and 62.86 in both eyes, n = 160). C. Mean RNFL thickness of group 3 (subjects in whom the corrected location point of the superior peak was ≥ 62.86 in 1 or both eyes, n = 56). D. The mean fractional interocular RNFL thickness differences. E. The mean absolute interocular RNFL thickness differences. [Straight line, right eye; dotted line, left eye; red line, group 1; black line, group 2; blue line, group 3; arrow heads, the mean locations of the superior peak RNFL thickness; gray zone, the area where the difference is statistically significant (P<0.05 Kruskal Wallis test)](TEMP = Temporal, SUP = Superior, NAS = Nasal, INF = Inferior).

The ocular information of the subgroups divided according to the location of the superior peak RNFL thickness is shown in Table 2. In subjects who had a more temporally located superior peak thickness in one or both eyes (group 1), both eyes exhibited a significantly lower SE, longer axial length, and lower signal strength compared to the other groups (P<0.05, Student’s *t* test). The mean RNFL thickness and the interocular differences of these 3 groups are shown in Fig. 5. The fractional interocular differences of the 3 groups exhibited significant differences at 11 points (4.30%, at points 47–52 and 58–62; P<0.05, Kruskal Wallis test). The subjects who had a more temporally located superior peak thickness (group 1) had a greater fractional interocular difference at superior area (at points 55–62) and a smaller interocular difference around the superior peak thickness (at points 47–49) than those subjects who had superior peak thickness within mean +/- 1SD (group 2) (P<0.05, Mann Whitney U test). The subjects who had a more nasally located superior peak thickness (group 3) had greater interocular difference at the temporal and superotemporal area (at points 9–11 and 46–57) and a smaller difference around the superior peak thickness (at points 63–64 and 118–119) compared to group 2 (P<0.05). The absolute interocular RNFL thickness differences of the three groups exhibited significant differences at 23 points (8.98%, at points 32–34, 73–79, 93–95 and 238–247, P<0.05). Group 1 had a greater interocular difference at the superotemporal and inferotemporal areas (at points 14–41 and 235–251) and a smaller interocular difference at the superonasal area (at points 91–96) compared to group 2 (P<0.05). Group 3 had a greater interocular difference at the superonasal area (at points 54–56 and 71–80) than group 2 (P<0.05).

**Table 2:** Ocular information of subgroups divided according to the location of the superior peak.

**Ocular variable**	**Group 1 (n = 44)**	**Group 2 (n = 160)**	**Group 3 (n = 56)**	P value*
Spherical equivalent of right eye (Diopter)	-4.102 ± 2.709	-2.098 ± 2.004	-1.752 ± 1.697	<0.001
Spherical equivalent of left eye (Diopter)	-4.074 ± 2.666	-2.108 ± 2.018	-1.623 ± 1.578	<0.001
Axial length of right eye (mm)	25.915 ± 1.331	24.676 ± 0.965	24.388 ± 1.127	<0.001
Axial length of left eye (mm)	25.886 ± 1.337	24.682 ± 0.989	24.381 ± 1.042	<0.001
Right eye’s corrected location of superior peak (point)	44.68 ± 4.75	55.08 ± 3.72	62.43 ± 5.24	<0.001
Left eye’s corrected location of superior peak (point)	46.66 ± 4.81	56.38 ± 3.27	65.70 ± 4.59	<0.001
Right eye’s corrected location of inferior peak (point)	215.43 ± 6.41	210.09 ± 7.26	205.54 ± 7.86	<0.001
Left eye’s corrected location of inferior peak (point)	215.70 ± 6.52	209.68 ± 7.26	205.96 ± 7.37	<0.001
Right eye’s average retinal nerve fiber layer thickness (μm)	98.18 ± 8.22	98.82 ± 9.05	99.84 ± 8.31	0.622
Left eye’s average retinal nerve fiber layer thickness (μm)	97.96 ± 8.48	98.11 ± 9.23	100.16 ± 8.25	0.299
Right eye’s signal strength of scan	8.18± 0.92	8.65 ± 0.946	8.86 ± 0.98	0.002
Left eye’s signal strength of scan	8.02 ± 1.00	8.53 ± 1.104	8.68± 0.92	0.005

(Group 1 is composed of subjects in whom the corrected location point of the superior peak was ≤ 48.78 in one or both eyes. Group 3 is composed of subjects in whom the corrected location point of the superior peak was ≥ 62.86 in one or both eyes. Group 2 is composed of subjects in whom the corrected location point of the superior peak was between 48.78 and 62.86 in both eyes.)

* By one-way ANOVA

**Fig 6:**
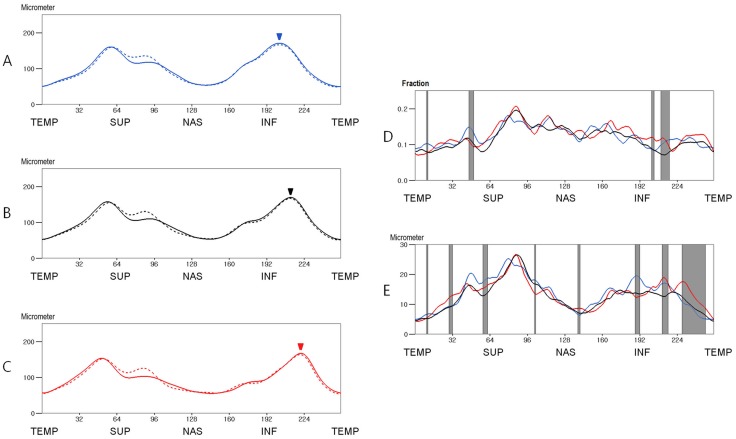
Mean RNFL thickness of subgroups classified by the location of the inferior peak thickness after correction for ocular cyclotorsion (A, B and C) and comparison of interocular RNFL thickness differences between the subgroups (D and E). A. Mean RNFL thickness of group 4 (subjects in whom the corrected location point of the inferior peak was ≤ 202.16 in 1 or both eyes, n = 62). B. Mean RNFL thickness of group 5 (subjects in whom the corrected location point of the inferior peak was between 202.16 and 217.75 in both eyes, n = 145). C. Mean RNFL thickness of group 6 (subjects in whom the corrected location point of the inferior peak was ≥ 217.75 in 1 or both eyes, n = 53). D. The mean fractional interocular RNFL thickness differences. E. The mean absolute interocular RNFL thickness differences. [Straight line, right eye; dotted line, left eye; red line, group 6; black line, group 5; blue line, group 4; arrow heads, the mean locations of the superior peak RNFL thickness; gray zone, the area where the difference is statistically significant (P<0.05 by Kruskal Wallis test)](TEMP = Temporal, SUP = Superior, NAS = Nasal, INF = Inferior).

The characteristics of the subgroups divided according to the location of the inferior peak RNFL thickness is shown in Table 3. Both eyes of the subjects in all 3 groups were significantly different in axial length (P<0.05, Student’s *t* test). In subjects who had a more temporally located inferior peak thickness (group 6), both eyes had a lower SE and a lower signal strength than the other groups (P<0.05). The mean RNFL thickness and the interocular differences of these 3 groups are shown in Fig. 6. The fractional interocular difference of the 3 groups exhibited significant difference at 20 points (7.81%; at points 10–11, 46–50, 114, 179, 202–204 and 210–217, P<0.05). The subjects who had a more temporally located inferior peak (group 6) had a greater fractional interocular difference at the inferotemporal area (at points 60–63, 113–115 and 202–216) than the subjects who had an inferior peak thickness within mean +/- 1SD (group 5) (P<0.05). The subjects who had a more nasally located inferior peak thickness (group 4) had a greater fractional interocular difference at the superotemporal and inferotemporal areas (at points 11–12, 44–52, 56–62, 114–116 and 209–222) than group 5 (P<0.05). The absolute interocular RNFL thickness differences of the 3 groups exhibited significant differences at 49 points (19.14%; at points 10–11, 29–32, 50, 58–62, 102–103, 139–141, 188–192, 211–216, and 228–248, P<0.05). Group 6 had a greater interocular difference at the inferotemporal area (at points 29–33, 209–215 and 227–251) than group 5 (P<0.05). Group 4 had a greater interocular difference at the superotemporal and interior areas (at points 47–63 and 115–116, 185–194 and 210–215) and a smaller interocular difference at the superotemporal area (at points 243–246) than group 5 (P<0.05).

**Table 3:** Ocular information of subgroups divided according to the location of the inferior peak.

**Ocular variable**	**Group 4 (n = 62)**	**Group 5 (n = 145)**	**Group 6 (n = 53)**	P value*
Right eye’s spherical equivalent of refractive error (Diopter)	-1.653 ± 1.470	-2.034± 1.958	-4.090 ± 2.734	<0.001
Left eye’s spherical equivalent of refractive error (Diopter)	-.1556 ± 1.511	-2.066±1.944	-3.988± 2.716	<0.001
Right eye’s axial length (mm)	24.393 ± 0.955	24.713 ± 1.096	25.630 ± 1.275	<0.001
Left eye’s axial length (mm)	24.402 ± 0.952	24.727 ± 1.112	25.568 ± 1.248	<0.001
Right eye’s corrected location of superior peak (point)	59.10 ± 6.88	55.23 ± 5.58	49.11 ± 6.51	<0.001
Left eye’s corrected location of superior peak (point)	60.66 ± 6.67	57.23 ± 5.83	50.81 ± 6.76	<0.001
Right eye’s corrected location of inferior peak (point)	200.68 ± 6.58	210.50 ± 3.98	219.60 ± 3.75	<0.001
Left eye’s corrected location of inferior peak (point)	200.63 ± 6.23	210.41 ± 4.04	219.36 ± 3.722	<0.001
Right eye’s average retinal nerve fiber layer thickness (μm)	100.44 ± 9.28	99.02 ± 8.64	96.93 ± 8.13	0.097
Left eye’s average retinal nerve fiber layer thickness (μm)	99.95 ± 9.09	98.44 ±9.03	97.12 ± 8.25	0.234
Right eye’s signal strength of scan	8.89 ± 0.87	8.62 ± 0.979	8.28 ± 0.97	0.004
Left eye’s signal strength of scan	8.81 ± 1.02	8.50 ± 1.06	8.04 ± 1.02	<0.001

(Group 4 is composed of subjects in whom the corrected location point of the inferior peak was ≤ 202.16 in one or both eyes. Group 6 is composed of subjects in whom the corrected location point of the inferior peak was ≥ 217.75 in one or both eyes. Group 5 is composed of subjects in whom the corrected location point of the inferior peak was between 202.16 and 217.75 in both eyes)

* By one-way ANOVA

## Discussion

In this study, the signed interocular average RNFL thickness difference value for the 2.5th and 97.5th percentile distributions are-8.14 μm and +8.00 μm, respectively. These values are similar to the results obtained by Mwanza et al. and are smaller than those obtained by Budenz et al. and Huynh et al [14–16]. Considering that we and Mwanza et al. used spectral domain OCTs and Budenz et al. and Huynh et al. used time-domain OCTs, these differences might be caused by different OCT machines [14–16].

Previously we investigated ocular factors affecting the RNFL symmetry score in normal adults and found that the symmetry score was strongly influenced by interocular difference in locations of the superior temporal retinal artery and vein and weakly influenced by interocular difference in axial length and refractive errors [12]. Further, the symmetry score was significantly increased after correction for ocular cyclotorsion. In the present study, the correction for ocular cyclotorsion decreased the regional interocular RNFL thickness. difference, the absolute interocular difference decreased by 10.11% and the fractional interocular RNFL thickness decreased by 7.89%. These findings suggest that the correction for ocular cylcotorsion enhances the interocular symmetry of the RNFL and the line connecting the foveola and the disc center can be a reliable reference line for interocular RNFL comparision.

The interocular RNFL thickness difference values exhibited variations according to the locations and comparison methods. The absolute interocular RNFL thickness difference values, which exhibited large variations according to locations and largely corresponded with RNFL thickness, were higher where RNFL thickness was great and lower where RNFL thickness was small. Although the average RNFL thickness of both eyes did not exhibit a significant difference (P = 0.104), the signed interocular RNFL thickness difference exhibited regional variations. The left eyes had a thicker RNFL around the superonasal area and the right eyes had a thicker RNFL around the temporal half of the RNFL. Because previous studies reported various results about the regional interocluar RNFL thickness differences [14–18], we are not sure about the above finding. This finding might be attributable to the selection bias caused by the enrollment of volunteers. Unlike the two comparison methods described above, the fractional interocular difference values exhibited relatively small variations according to the locations. The fractional interocular differences in the nasal half were slightly greater than those of temporal half (in temporal half, 0.991 ± 0.011; in nasal half, 0.144 ± 0.019; Fig. 3A), and the tolerance limits for the 95th and 99th distributions in the nasal half were also greater than those of temporal half area (0.242 ± 0.017 and 0.364 ± 0.040 versus 0.340 ± 0.032 and 0.437 ± 0.048, respectively; Fig. 3A).

The present study reported the 95th and 99th percentile distributions for the interocular RNFL thickness difference at each measurements points. We consulted a statistician and calculated the probabilities that the interocular RNFL thickness differences would exceed the 99th percentile distributions at consecutive points by using formulas for correlated events [19]. According to the probabilities calculated by Frank’s model for correlated events, 0.25% of normal subjects would have 3 consecutive points where the fractional interocular difference exceeds the 99 percentile distribution, and only 0.56% of normal subjects would have 5 consecutive points where the fractional interocular difference exceeds the 99 percentile distribution. Thus, glaucoma can be strongly suspected if the interocular difference values exceed the 99th percentile distributions at more than 3 consecutive points or the 95th percentile distribution at more than 5 consecutive points.

This study evaluated the interocular RNFL thickness difference in different ways. Among these methods, we propose that evaluation of fractional interocular difference is the most suitable mechanism for evaluating the interocular RNFL thickness difference. First, the fractional interocular difference is relatively free from the ocular magnification effect. Most individuals do not have significant anisometropia and the right and left eyes have a similar ocular dimension and refractive status. If both eyes have similar ocular dimensions and refractive statuses, the observed RNFL thickness of both eyes and the interocular RNFL thickness difference would be influenced by a similar magnitude of ocular magnification. Therefore, the ocular magnification effect would be minimized if the thickness difference were divided by the observed RNFL thickness. As shown in this study, individuals who have deviated RNFL thickness profiles usually differ from other individuals in axial length and SE, which indicates that observed RNFL thickness should be corrected for ocular magnification effects for correct comparisons. Thus, the fractional interocular RNFL thickness comparison has an advantage over simple interocular RNFL thickness comparison especially for the individuals with deviated RNFL thickness profiles. Second, the tolerance limits for the 95th and 99th distributions in the fractional interocular RNFL thickness difference were relatively constant and exhibited less variation than the absolute interocular difference (Fig. 4). Thus, among the mechanisms for determining the interocular RNFL thickness difference, developing a normative range for the fractional interocular RNFL thickness difference value would be simplest and its clinical application would be easiest. Third, when comparing the interocular RNFL thickness difference between the subjects with deviated RNFL thickness profiles and the subjects with non-deviated RNFL thickness profiles, comparison using the fractional interocular difference exhibited differences at smaller area than comparison using the absolute interocular difference (4.30% versus 8.98% and 7.81% versus 19.14%, P = 0.049 and P <0.001 by chi-square, respectively). This finding suggests that the fractional interocular differences of individuals with deviated RNFL thickness profiles are not considerably different from those of individuals with non-deviated RNFL thickness profiles; thus, the fractional interocular difference values of individuals with non-deviated RNFL thickness profiles can be applied to individuals with deviated RNFL thickness profiles.

This study had some limitations. First, all of the subjects were very young. Previous studies have reported a negative correlation between RNFL thickness and age [20–23], suggesting that the physiologic aging process may affect interocular RNFL thickness symmetry. Second, our results may not be applicable to patients of all ethnic backgrounds because all of our subjects were Korean. However, because Mwanza et al. reported similar results regarding interocular average RNFL thickness differences in subjects with different ages and ethnicities [14], the interocular RNFL thickness difference may not be significantly influenced by ethnicity or age. Finally, because our study group did not contain subjects with anisometropia over 1.5 D or an interocular axial length difference over 0.3 mm, our results may not be applicable to patients with significant anisometropia.

Although glaucoma usually affects both eyes, it is often asymmetric at presentation and during progression. Thus, we think that the interocular RNFL thickness comparison would be a very efficient glaucoma diagnostic method except in very rare cases in whom glaucoma affects both eyes at the same RNFL location and to the same extent. Further, we recommend to use the fractional interocular comparison method, which has many advantages over the simple interocular comparison. Glaucoma can be strongly suspected, if interocular fractional RNFL thickness differences of a patient are over 25% at more than 5 consecutive points or over 35% at more than 3 points in the temporal half of peripapillary area. We believe that the fractional interocular RNFL thickness comparison is promising as a diagnostic tool, and we hope that the results of our study will facilitate the diagnosis of early glaucoma.
